# Transcriptomics Provide Insights into Early Responses to Sucrose Signaling in *Lupinus albus*, a Model Plant for Adaptations to Phosphorus and Iron Deficiency

**DOI:** 10.3390/ijms25147692

**Published:** 2024-07-13

**Authors:** Tahmina Shammi, Yishen Lee, Jayati Trivedi, Dakota Sierras, Aniqua Mansoor, Jason M. Maxwell, Matthew Williamson, Mark McMillan, Indrani Chakravarty, Claudia Uhde-Stone

**Affiliations:** Department of Biological Sciences, California State University, East Bay, Hayward, CA 94542, USA; tshammi@horizon.csueastbay.edu (T.S.);

**Keywords:** iron deficiency, nanopore sequencing, phosphate deficiency, sucrose signaling, white lupin

## Abstract

Phosphorus (P) and iron (Fe) deficiency are major limiting factors for plant productivity worldwide. White lupin (*Lupinus albus* L.) has become a model plant for understanding plant adaptations to P and Fe deficiency, because of its ability to form cluster roots, bottle-brush-like root structures play an important role in the uptake of P and Fe from soil. However, little is known about the signaling pathways involved in sensing and responding to P and Fe deficiency. Sucrose, sent in increased concentrations from the shoot to the root, has been identified as a long-distance signal of both P and Fe deficiency. To unravel the responses to sucrose as a signal, we performed Oxford Nanopore cDNA sequencing of white lupin roots treated with sucrose for 10, 15, or 20 min compared to untreated controls. We identified a set of 17 genes, including 2 bHLH transcription factors, that were up-regulated at all three time points of sucrose treatment. GO (gene ontology) analysis revealed enrichment of auxin and gibberellin responses as early as 10 min after sucrose addition, as well as the emerging of ethylene responses at 20 min of sucrose treatment, indicating a sequential involvement of these hormones in plant responses to sucrose.

## 1. Introduction

Phosphorus (P) is an essential mineral nutrient required for plant growth and development and plays important roles in cellular processes such as macromolecule synthesis, energy storage, and signal transduction [1]. P deficiency in soils is a global problem with significant implications for long-term crop sustainability, exacerbated by the misuse of rock phosphate fertilizers [2]. To find solutions, researchers are learning from plants that are well adapted to nutrient deficiencies, such as white lupin (*Lupinus albus*), which has become an illuminating model for the study of plant adaptations to P and iron (Fe) deficiency [3,4]. Under P and Fe deficiency, white lupin forms cluster roots, specialized roots that resemble bottle brushes and that allow white lupin to acquire nutrients unavailable to most other plants [5]. White lupin’s unique adaptations to P deficiency, such as the development of cluster roots to augment the root surface area, have been elucidated through physiological studies [6,7,8,9,10]) and transcriptomics [4,11,12,13,14].

However, the signaling pathways involved in white lupin (or other plants) to sense P deficiency and to elicit responses are not thoroughly understood. Split-root experiments in white lupin have revealed that P deficiency is sensed in the shoot and communicated to the root [15]. This realization started a search to identify the long-distance signal that is transported from shoot to root in response to P deficiency. This research revealed that under P deficiency, microRNA 399 and an increased concentration of sucrose were transported via the phloem to the root, where miR399 targeted PHO2 mRNA, a ubiquitin E2-conjugating enzyme that acts as an inhibitor of the phosphate starvation response [16,17,18]. In white lupin, miRNA399 was not induced under P deficiency in the dark or after stem girdling, indicating that phloem transport of photosynthates, most likely in the form of sucrose, are a requirement for miR399 shoot-to-root signaling [19].

Several studies in recent decades have revealed that sucrose acts not only as a metabolite, but also as a major long-distance signal sent from the shoot to the root to signal P and Fe deficiency [20,21]. In white lupin and Arabidopsis, increased sucrose flux from shoots to roots coincides with changes in root morphology and architecture, resulting in reduced primary root and increased lateral root growth, which increases the surface area to more efficiently mine the soil for scarce nutrients [3,19]. Studies in white lupin have indicated that exogenously supplied sugars and photosynthates stimulate the transcription of several genes involved in cluster root functioning, such as LaPT1 (*Lupinus albus* phosphate transporter 1), *LaSAP* (secreted acid phosphatase) and *LaMATE* (multi-drug and toxin extrusion), indicating that sugar signaling induces the expression of P starvation-responsive genes [19]. 

Later studies showed that sucrose added to the growth medium was able to induce cluster root formation in white lupin [22,23], which usually occurs only in response to P and Fe deficiency. One of these studies [23] indicated that the expression of some P-responsive genes, including LaPT1, was increased by a combination of P limitation and sucrose addition, while the expression of LaSAP was stimulated by sucrose independently of P supply [23]. However, a later study on sucrose-induced cluster roots revealed that externally added sucrose, while triggering cluster root formation, did not result in cluster root functioning, and the expressions of P-responsive genes such as LaSAP and LaMATE were not up-regulated in sucrose-induced cluster roots [22]. These findings indicate that sugars other than sucrose may regulate cluster root function. Recently, trehalose was shown to be involved in both the formation and function of cluster roots [24]. 

The hypothesis that sucrose acts as a signal of nutrient deficiency is further supported by studies that have revealed a role of sucrose in the signaling of other nutrient deficiencies besides P and Fe, including nitrogen (N) and potassium (K) [25]. In soybean, RNA-seq has revealed that sucrose added to the root for 20 or 40 min induces the expression of genes involved in various nutrient deficiencies, particularly those of Fe and N [26]. The somewhat surprising finding that sucrose is involved in the signaling of different nutrient deficiencies may explain the well-known observation of crosstalk, an overlap of signaling pathways and plant responses to various nutrient deficiencies [27]. Indeed, several genes identified as up-regulated in cluster roots of white lupin under P deficiency are also up-regulated in cluster roots under Fe and N deficiency [28,29] while other P-responsive genes are specific to P deficiency [28]. Taken together, these findings indicate that sucrose induces more general stress responses, while other signals, such as microRNAs, may play a role in triggering specific nutrient stress responses [3,30]. 

In addition to a role of sucrose in abiotic stress responses, several studies have revealed a role of sucrose signaling in response to biotic stresses [31,32]. The exogenous application of sucrose, for example, has been shown to induce the expression of defense-related transcription factors in rice [33]. In soybean, genes involved in biotic stresses were enriched among up-regulated genes after the short-term (20 and 40 min) addition of sucrose to the roots [26]. Taken together, many genes in response to biotic and abiotic stresses are induced by sucrose, supporting a role of sucrose as a signaling molecule [34].

RNA-seq is a well-established approach to assess differential gene expression, e.g., in response to nutrient deficiencies [4]. The use of Oxford Nanopore Technologies is making RNA-seq more affordable and enabling the sequencing of longer reads, which is helpful for mapping and for distinguishing splice variants.

Our current study focuses on using Oxford Nanopore cDNA sequencing of white lupin roots grown hydroponically with sufficient nutrients. After three weeks, plant roots were treated with external sucrose added directly into the hydroponics solution for 0 (control), 10, 15, or 20 min. Our goal was to identify early key contributors within the sucrose signaling pathway, giving deeper insight into how white lupin plants respond to sucrose signaling. In the long term, a better understanding of sucrose signaling could help in the development of plants with increased tolerance to biotic and abiotic stresses.

## 2. Results

### 2.1. Nanopore cDNA Sequencing to Assess Short-Term Responses to Sucrose Resulted in 35.5 Million Reads

To mimic the sucrose signal that is transported from the shoot to the root in response to P and Fe deficiency, we added sucrose directly to the roots of hydroponically grown white lupin. We decided to add external sucrose at a concentration of 10 mM based on previous studies that have shown that cluster roots in white lupin can be mimicked by adding sucrose to the growth medium at a concentration range of 2.5 to 12.5 mM, while a further increase to 25 mM sucrose leads to unusual root thickening [22]. This data are in line with the concentration of internal sucrose at the cluster root initiation zone, which was measured at 3.4 mM sucrose [22].

After hydroponic growth in full nutrient solution for three weeks, roots were subjected to external sucrose at a final concentration of 10 mM for 0 (control), 10, 15, or 20 min in three biological replications for a total of twelve samples. Twelve corresponding cDNA libraries were combined into three pools, one for each biological replication, and each biological replication was sequenced on a different Oxford Nanopore Minion flow cell, resulting in an initial 35,545,919 total reads.

To further analyze our data, we used the Epi2me wf-transcriptomes workflow available at https://github.com/epi2me-labs/wf-transcriptomes, accessed on 1 May 2024. In this workflow, Pychopper (https://github.com/nanoporetech/pychopper, accessed on 26 April 2024) was used to remove adapters and low-quality read portions. Minimap2 was used to map sequence reads to the *Lupinus albus* reference genome CNRS_Lalb_1.0 (GCA_009771035.1 assembly; submitted. 20 December 2019) [35]. This resulted in a total of 20,879,968 mapped reads corresponding to 24,655 *L. albus* genes (Table 1), which represent about 64% of the currently annotated 38,255 protein-coding genes in the white lupin reference genome (https://www.ncbi.nlm.nih.gov/datasets/genome/GCA_009771035.1/, accessed on 25 April 2024).

### 2.2. A Set of 17 Genes Was Up-Regulated at All Three Time Points of Sucrose Exposure 

**Figure 1:**
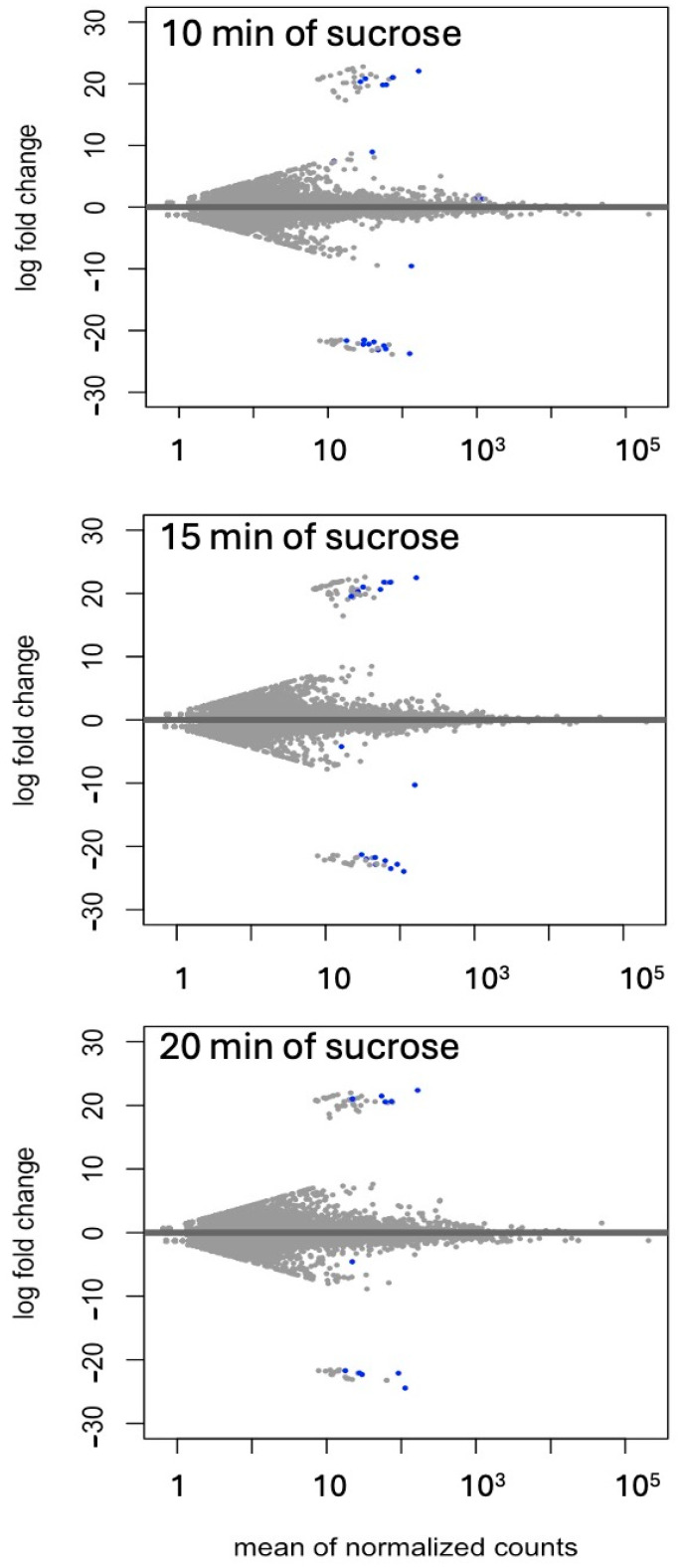
MA (mean average) plot of log2 FC against normalized transcript counts at 10, 15, and 20 min of sucrose treatment, each compared to no-sucrose control. Each time point is based on three biological replications. Values of padj (adjusted *p*-value) ≤ 0.01 in the DESeq2 time-course expression analysis are shown in blue.

**Figure 2:**
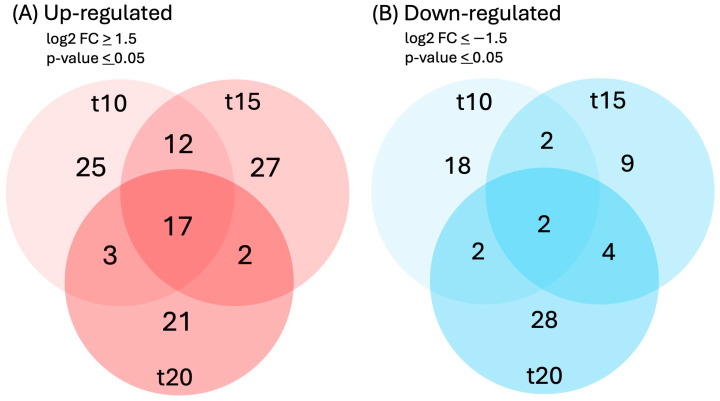
Venn diagram of genes that were (**A**) up-regulated (log2FC ≥ 1.5, *p*-value ≤ 0.05) or (**B**) down-regulated (log2FC ≤ −1.5, *p*-value ≤ 0.05) in response to 10, 15, or 20 min of sucrose treatment.

Differential expressions of genes and transcripts were further analyzed with DESeq2 [36]. MA (mean average) plots (Figure 1) were used to visualize the resulting log2 FC (fold change) against normalized sequence counts at 10, 15, and 20 min of sucrose exposure, each compared to 0 min (control), revealing that some differential gene expression had already occurred at 10 min after sucrose addition. A PCA (principal component analysis) plot did not reveal clear differences between the four time points used in this study, likely due to the relatively few changes at such short exposure. While longer exposure and larger differences between time points would likely reveal larger numbers of differentially expressed genes and better separation between time points, we were interested in the earliest responses to sucrose to identify potential key players in the responses to sucrose. A Venn diagram (Figure 2) revealed a set of 17 genes that were up-regulated at all three time points of sucrose exposure. 

### 2.3. Auxin- and Gibberellin-Responsive Genes and Two bHLH Transcription Factors Are among the Earliest Up-Regulated Genes

Table 2 shows a set of 17 genes that were up-regulated at all three time points of sucrose treatment. Two of these genes were involved with the plant hormone auxin: “small auxin-up RNA” is an auxin-induced protein of unknown function, while WAT1 is a vacuolar auxin transporter. Among the 17 up-regulated genes were also two gibberellin-responsive proteins. Because we were interested in key regulators of early responses to sucrose, we were especially interested in the two basic helix–loop–helix transcription factors. Expansin and xyloglucan endotransglucosylase/hydrolase, both involved in cell wall organization, were also up-regulated, as was defensin, part of biotic stress responses.

**Figure 3:**
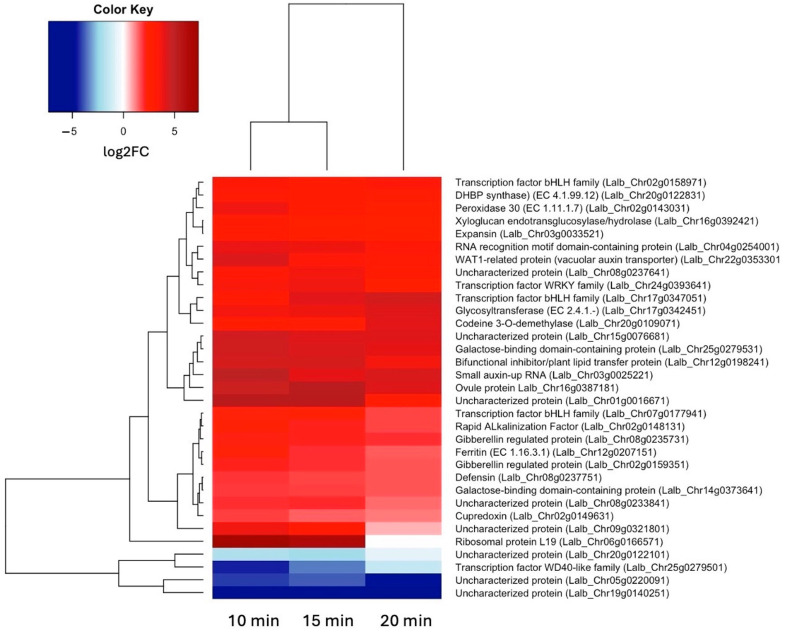
Heatmap showing hierarchical clustering of the 29 genes that were up-regulated (log2FC ≥ 1.5, *p*-value ≤ 0.05) and 4 genes that were down-regulated (log2FC ≤ –1.5, *p*-value ≤ 0.05) at both 10 and 15 min of sucrose exposure.

Because we are particularly interested in early responses to sucrose, we also looked at all genes that were up- or down-regulated at both 10 and 15 min of sucrose exposure, encompassing 29 up-regulated and 4 down-regulated genes (Figure 3). In addition to the two bHLH transcription factors, a WRKY transcription factor showed significant up-regulation at these early time points. 

**Figure 4:**
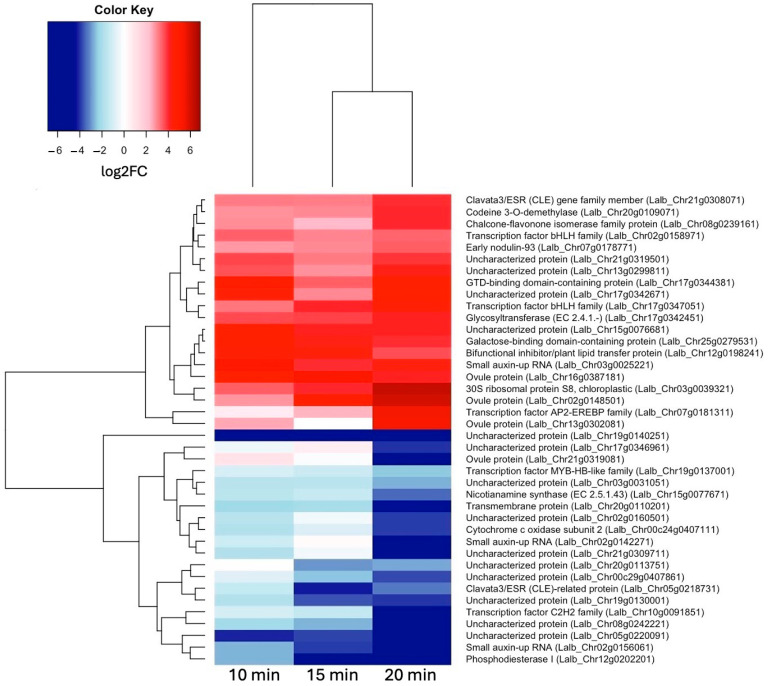
Heatmap showing hierarchical clustering of 20 most up-regulated genes (log2FC ≥ 1.5, *p*-value ≤ 0.05) and 20 most down-regulated genes (log2FC ≤ –1.5, *p*-value ≤ 0.05) at 20 min of sucrose treatment.

To see how these early genes may differ from genes activated slightly later, we also looked at the most up- and down-regulated genes at 20 min of sucrose treatment (Figure 4). Interestingly, Clavata3/ESR (CLE) homologs were among the most up- and most down-regulated genes at 20 min of sucrose exposure. A Blast search revealed that the up-regulated CLE gene was most similar to CLE44, and the down-regulated CLE gene was most similar to CLE4. Also worth pointing out is the AP2-EREB (APETALA2-ethylene-responsive element-binding protein) family-type transcription factor, which became more up-regulated with the increasing sucrose exposure time.

### 2.4. qRT-PCR Confirms Differential Expression of Selected Genes

**Figure 5:**
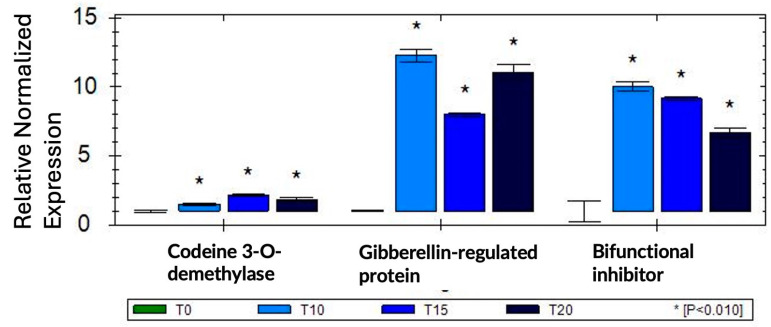
qRT-PCR validation of three selected sucrose-responsive genes in one biological replication; asterisks are based on SEM (standard error of mean) across three technical replications.

To validate our RNA-seq results, we selected three genes (codein 3-O demethylase, gibberellin-regulated protein, and bifunctional inhibitor/plant lipid transfer protein/seed storage helical-domain-containing protein) that were up-regulated in response to sucrose exposure and tested these genes using qRT-PCR in one biological and three technical replications. To normalize gene expression, we used two reference genes (Histone H2A variant3 and proteasome endopeptidase complex) that we identified from a set of four potential reference genes as the most stable in response to sucrose using geNorm [37]. The qRT-PCR results confirmed our RNA-seq data, and all three genes showed up-regulation in response to sucrose (Figure 5).

### 2.5. GO Analysis Reveals Enrichment of Sugar- and Hormone-Responsive Genes 

**Figure 6:**
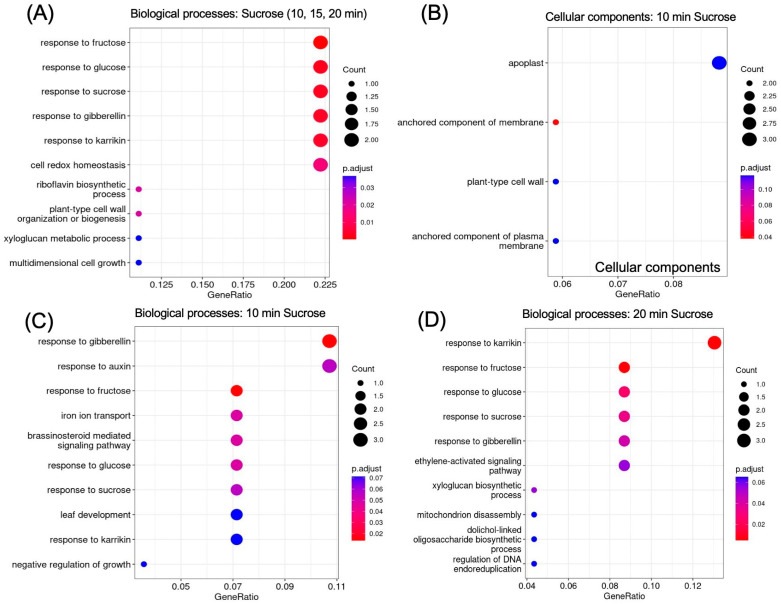
GO enrichment of up-regulated genes (log2FC ≥ 1.5, *p*-value ≤ 0.05). (**A**) Biological processes enriched in genes up-regulated at all time points of sucrose treatment (t10 AND t15 AND t20). (**B**) Cellular components enriched 10 min after sucrose addition. (**C**) Biological processes enriched at 10 min and (**D**) at 20 min of sucrose exposure.

GO (gene ontology) enrichment revealed responses to hormones and to sucrose as enriched biological processes (Figure 6A). The apoplast, cell wall, and membrane were revealed as enriched locations (Figure 6B). To further delineate the timeline of biological processes that are activated in response to sucrose, we looked separately at enrichment at 10 min (Figure 6C) and 20 min (Figure 6D) of sucrose addition. At 10 min of sucrose exposure, responses to the plant hormone gibberellin were most significantly enriched; other responses included responses to auxin, brassinosteroids, and sucrose. In regard to nutrient deficiency signaling, it is of interest that iron ion transport was also an enriched term. At 20 min, the ethylene-activated signaling pathway became enriched, revealing a possible involvement of this signaling pathway later in the sucrose response.

### 2.6. Several Sucrose-Induced Genes Are also Expressed in Cluster Roots

**Figure 7:**
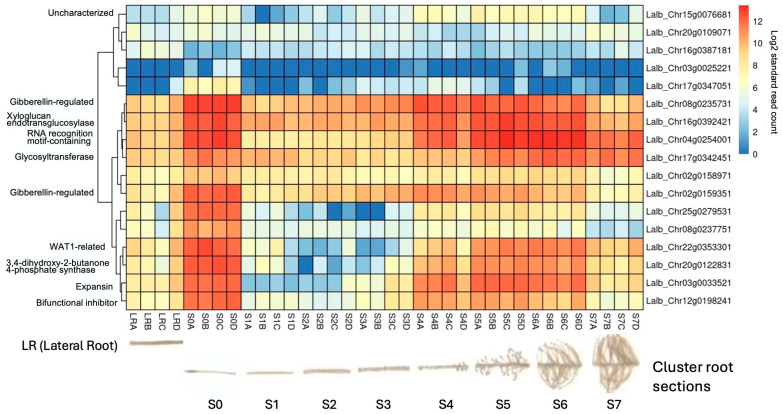
Hierarchical clustering of the 17 shared up-regulated genes using publicly available gene expression data [35] revealed that 10/17 (59%) were also induced in white lupin cluster roots, particularly in root tip section (S0) and just-emerging (S4), premature (S5), and mature (S6) cluster root sections, but not in early root sections S1–S3 nor in older cluster root sections (S7). The letters after the section numbers denote four biological replications (A–D); the colors indicate log2-transformed normalized reads.

As the molecular mechanisms that control cluster root formation remain unknown, the search for key regulators in cluster root development is of great interest. Because external application of sucrose can induce cluster roots in white lupin [22,23], we were interested in identifying genes in our data set that were up-regulated in response to sucrose and were also differentially expressed in cluster roots. Using the gene expression profile tool of the *Lupinus albus* Genome Browser [35], we performed hierarchical cluster analysis of the 17 genes which were up-regulated in response to sucrose treatment at all three time points (log2FC ≥ 1.5, *p*-value ≤ 0.05). This analysis identified 10/17 genes that were indeed also up-regulated in cluster root sections compared to regular lateral roots (Figure 7). These included the two gibberellin-regulated proteins and a WAT1-related gene (vacuolar auxin transporter). Cluster root sections that showed the most up-regulation included S4 (just emerging), S5 (premature), and S6 (mature) cluster roots.

## 3. Discussion

### 3.1. A Network of Sucrose-Responsive Genes Is Involved in Cell Growth and Differentiation

Plants have evolved a coordinated response to P deficiency that is tightly coupled with carbon (C) assimilation and allocation. Under P or Fe deficiency, increased allocation of C (mainly in form of sucrose) optimizes root growth toward a higher root-to-shoot ratio and a changed root architecture with increased lateral root growth [30,38,39]. In addition, an increase in sucrose transported from the shoot to the roots also acts as a long-distance signal for P [20] and Fe [40] deficiency. White lupin has become a model plant for adaptations to P and Fe deficiency because of its ability to form cluster roots, bottle-brush-like root structurers that enhance P and Fe solubilization and uptake [3,4,41]. Interestingly, the formation of these cluster roots can be triggered by external sucrose application [22,23]. 

We were interested in unraveling the regulatory network that becomes activated in the root in response to sucrose signaling. To mimic the sucrose signal, we added sucrose directly to the roots of hydroponically grown lupin at a concentration (10 mM) known to trigger cluster root formation in white lupin [22]. Oxford Nanopore sequencing of cDNA proved useful as a method to look at the global gene expression changes in response to such short-term sucrose addition to the roots. Our results revealed significant up- and down-regulation already at 10 min of sucrose addition, as well as a set of 17 genes that were up-regulated at all three time points (10, 15 and 20 min) of sucrose exposure.

**Figure 8:**
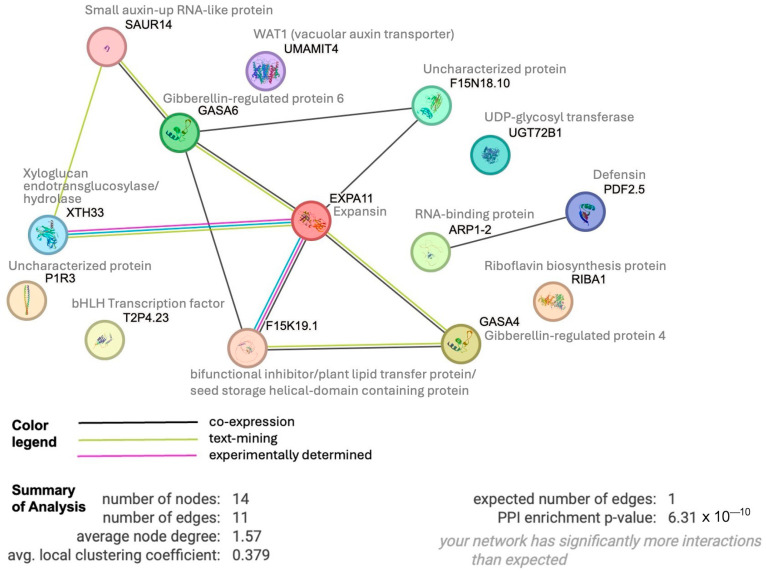
Protein interactome generated by STRING analysis (https://string-db.org) of 14 Arabidopsis homologs of our set of 17 shared up-regulated white lupin genes, revealing significant enrichment of interactions (*p*-value 6.3^−10^). Edges indicate possible connections based on co-expression (black), text mining (green) or experiments (magenta).

To determine whether these 17 up-regulated genes shared any connections, we performed protein interactome analysis [42] of the 14 Arabidopsis paralogues we could identify. This analysis revealed an enrichment (*p*-value 6^−10^) of protein–protein associations between these 14 homologs (Figure 8), indicating relevant biological connections. This interactome depicts expansin as a central hub around which many interactions center. Expansins, xyloglucan endotransglucosylase/hydrolase, and SAUR (SMALL AUXIN UP-REGULATED RNA) proteins are all involved in auxin-induced cell growth [43]. WAT1 (WALLS ARE THIN1), while not connected in the interactome, is a vacuolar auxin transport facilitator and has been shown to be important for maintaining cell wall thickness [44]. These findings indicate a role of auxin in short-term sucrose responses, which is in line with earlier suggestions of sucrose increasing root responsiveness to auxin to promote lateral root and root hair formation during P deficiency [45]. Our findings are further supported by recent findings in Arabidopsis that demonstrate sucrose acting as a long-distance signal and regulating the local biosynthesis of auxin at the primary root tip [46]. 

In the interactome, expansin also shows a connection to a bifunctional inhibitor/plant lipid transfer protein/seed storage (BI/LTP/SS) helical domain-containing protein. Proteins with this domain are typically lipid transfer proteins located in the cell wall and can be involved in key cellular processes, such as the stabilization of membranes, cell wall organization, and signal transduction [47,48]. It is worth noting that two other BI/LTP/SS helical domain-containing proteins were also significantly up-regulated at 10 min of sucrose exposure, indicating an important role of members of this protein family in sucrose responses. Defensins, part of our set of 17 up-regulated genes, are also located in the cell wall and extracellular space. They can be induced by pathogen attack, wounding, and some abiotic stresses [44]. To confirm the apoplastic localization of BI/LTP/SS and defensin, we used DeepLoc 2 [49], which indeed identified signal peptides for these proteins and predicted their likely location as extracellular.

Among the 17 shared up-regulated genes were 2 bHLH transcription factors. One of these showed the highest homology to the bHLH transcription factor UPBEAT1 (UPB1), known to regulate the expression of a set of peroxidases, which in turn modulate the balance of reactive oxygen species (ROS) in the root [50]. ROS signaling can activate other responses and determine the transition between the meristematic and elongation zones of roots. We looked for differentially expressed peroxidases and, indeed, found one peroxidase (Lalb_Chr02g0143031) to be up-regulated already at 10 min of sucrose exposure. 

Another type of meristem regulators, Clavata3/ESR (CLE) paralogues were among the most up- and down-regulated genes at 20 min of sucrose exposure. It is worth noting that the up-regulated and down-regulated genes encode different CLE peptides (CLE 44- and CLE 4-like). CLEs are peptide signals, often transported in the xylem or phloem and shown to bind to leucine-rich repeat receptor-like kinases, though the mechanisms of signal transduction are still largely unknown [51]. Interestingly, mutant analyses have shown that CLE genes positively affect the root sucrose level [52]. CLEs are known to influence root architecture in other plants [53], and a possible role of CLE in fine-tuning white lupin cluster root development has recently been suggested [54]. 

### 3.2. Timing and Coordination of Sucrose Responses

GO (gene ontology) enrichment comparing 10 and 20 min of sucrose exposure in our study displayed much overlap, but also a few interesting differences between time points, which may help to delineate the timeline of sucrose responses. At 10 min of sucrose treatment, responses to auxin and gibberellin were the most enriched biological processes, while responses to ethylene were enriched only at 20 min, indicating that ethylene-mediated responses may act later in the network of sucrose signaling. This finding supports our recent work on sucrose responses in soybean (20 and 40 min of sucrose exposure), where responses to ethylene were highly enriched 40 min after sucrose addition [26]. The study in soybean plants found an increase in ROS signaling and Ca^2+^ signaling at 40 min of sucrose exposure, but we were unable to determine the order (does ROS signaling lead to Ca^2+^ signaling, or vice versa?). Our current study in white lupin showed enrichment of REDOX events and up-regulation of a peroxidase at 10 min of sucrose treatment, indicating that ROS signaling may occur early in the response to sucrose. We did not find any evidence for Ca^2+^ signaling in our short-term study, indicating that Ca^2+^ signaling—if involved in the sucrose response in white lupin—may occur later. 

While our previous study in soybean identified many transcriptional factors, our current study on even earlier time points was successful in narrowing down the number of transcriptional regulators to a total of four up-regulated transcription factors and one down-regulated transcription factor. Two bHLH transcription factors mentioned above were up-regulated at all three time points, while an AP2-EREB (APETALA2-ethylene-responsive element-binding protein) family-type transcription factor was up-regulated at 20 min of sucrose exposure. Interestingly, one WRKY transcription factor was only up-regulated at 10 (highest) and 15 min of sucrose exposure, indicating possible involvement very early in the sucrose response. Another gene of interest that was upregulated early (10 and 15 min of sucrose) was a rapid alkalinization factor (RALF), which belong to peptide hormones that control cell wall integrity and cell-to-cell communication and can act as sensors for regulating responses to environmental stimuli [55]. Thus, they may play a central part in regulating sucrose responses. 

A previous microarray study on Arabidopsis leaves [56] compared the responses to P starvation (4 weeks) and sucrose treatment (leaves soaked with 100 mM sucrose for 16 h) and found that 6.1% of genes responded to P starvation, 25.5% to sucrose treatment, and only 0.7% to both factors (0.7%), indicating that sucrose signaling goes beyond P starvation responses and that most P-responding genes were independent from sucrose treatment. The relatively small overlap between P deficiency and sucrose responses found in that study may be due in part to the differences in timing, i.e., 4 weeks of P deficiency versus 16 h of sucrose treatment. Indeed, another transcriptome study in Arabidopsis [57] indicated at least two transcriptional programs operating in response to P starvation; 4 h of P deficiency activated more general stress response genes, while after 100 h, genes with more specific roles in the P starvation response became induced.

Recent RNA-seq experiments on Zygnematophyceae—the closest algal relatives of land plants—exposed to light and heat stress, in combination with extensive data mining of stress response experiments in true plants, have identified conserved stress hubs common to both algae and plants, indicating that these originated before plants moved to land [58]. We found some components of these general stress response hubs that were also differentially expressed in our study, including genes related to ROS metabolism, cell wall maintenance, and certain plant hormones, such as ethylene response factors. Other common stress response genes involving abscisic acid (ABA) signaling or mitogen-activated protein kinases (MAPK) were not upregulated in our study, possibly due to the facts that we looked only at the earliest responses. In addition, genes involved in plastid–nucleus communication and light responses, also part of the general plant stress hubs, were not enriched in our study, likely due to the fact that we focused on roots, where chloroplasts and light responses play less of a role.

**Figure 9:**
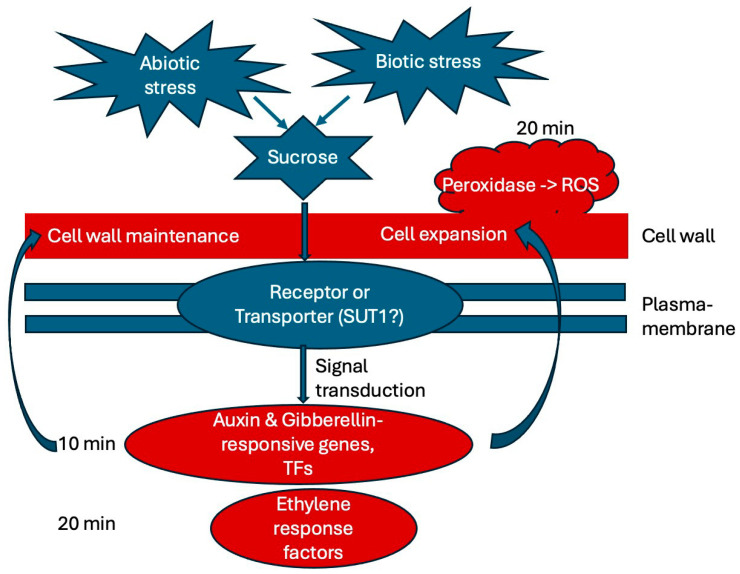
Working model summarizing the main findings of this study. External sucrose binds to a receptor or SUT (sucrose transporter) and triggers signal transduction pathways. Our results indicate that auxin and gibberellin are involved in the earliest (10 min) responses to sucrose exposure, while ethylene response factors and peroxidase are up-regulated at 20 min of sucrose treatment. Cell wall maintenance and cell expansion are among the most noticeable responses. Abbreviations: SUT (sucrose transporter), SnRK1 (sucrose-nonfermenting1–related kinase 1), ROS (reactive oxygen species), TFs (transcription factors).

We summarized our findings in a working model (Figure 9), showing what is suggested from the literature in blue and main finding of this study in red, delineating an initial timeline of early responses to sucrose. In the future, it would be interesting to further analyze the potential function of the identified transcription factors and the rapid alkalinization factor in response to sucrose. We are also interested in looking at even earlier time points of sucrose exposure to identify the very earliest responses, which may identify one or a few key regulators. We are also interested in analyzing later time points of white lupin’s responses to sucrose to reveal whether responses to biotic stresses become enriched, as is the case in soybean [26]. Unraveling the complex network of sucrose responses in plants will be helpful in order to better understand how plants integrate various nutrient deficiencies as well as other abiotic and even biotic stress responses, using sucrose not only as a metabolite, but also as a signal.

## 4. Materials and Methods

### 4.1. Plant Growth and Treatments

White lupin (*Lupinus albus* cv. Amiga) seeds were sterilized by shaking for 3 min in 10% bleach, followed by several rinses with sterile water. Sterile seeds were then spread out in sterile petri dishes and covered about halfway with sterile water to germinate at room temperature in the dark for 3–4 days. Once the radicles had reached a length of 2–3 cm, the seedlings were transferred to hydroponics containers filled with 850 mL of Hoagland solution [59], which was changed about every 4 days. The temperature of the growth chamber was maintained at ~21 °C with a light cycle of 16 h and a dark cycle of 8 h [60]. 

After 21 days of cultivation in hydroponics, the plant roots were exposed to sucrose by adding 8.5 mL of 1 M sucrose (prepared in Hoagland solution) directly to the hydroponic solution for a final concentration of 10 mM sucrose. Harvesting was carried out after exposing the plants to sucrose for different periods: 0 (control), 10, 15, or 20 min. All time points were assessed in 3 biological replications; each biological replication consisted of one plant. For harvesting, about 100 mg of root tip sections with lengths of 5–6 cm were harvested in liquid nitrogen from each plant and stored immediately at −80 °C.

### 4.2. RNA Isolation and Quality Check 

RNA from white lupin samples was isolated following the protocol for “Purification of Total RNA from Plant Cells and Tissues, and Filamentous fungi” from the RNasy Plant Mini kit (Qiagen, Valencia, CA, USA). The Qubit 4 Fluorometer (Thermo Fisher Scientific, Waltham, MA, USA), in conjunction with an RNA-high-sensitivity assay (Thermo Fisher Scientific, Waltham, MA, USA), was used to assess RNA quantity. In addition, the RNA IQ assay was used to determine the RNA integrity number (RIN). This RIN was based on the ratio of large and/or structured RNA to small RNA in the sample. Only samples with RINs of 8 or higher were used for RNA sequencing.

### 4.3. cDNA Library Preparation and RNA-Sequencing 

Using the PCR-cDNA sequencing-barcoding (SQK-PCB111) kit of Oxford Nanopore Technologies, the extracted RNA was converted into cDNA and uniquely barcoded following the manufacturer’s instructions. Equal concentrations of the four cDNA libraries for each biological replication were pooled and sequenced on three separate MinION FLO-MIN106 flow cells (Oxford Nanopore Technology, Lexington, MA, USA) using a MinION MK1C running Minknow v20.06.5 and guppy v4.09. Basecalling was performed during the run using the fast-basecalling algorithm with a Q score cutoff > 7. 

### 4.4. RNA-seq Data Analysis

Demultiplexed sequencing reads in fastq format were transferred from the Mk1C device to a PC with Epi2me installed. The data were analyzed using the Epi2me wf-transcriptomes workflow version 1.1., accessed on 26 April 2024, available at https://github.com/epi2me-labs/wf-transcriptomes. This pipeline consisted of the following steps: First, fastcat was used to concatenate files and generate read statistics, followed by Pychopper to orient, trim, and rescue full-length cDNA reads. Then, Minimap2 was used to map reads to the *Lupinus albus* reference genome CNRS_Lalb_1.0 (GCA_009771035.1 assembly; submitted. 20 December 2019, (Hufnagel, Marques et al. 2020 [39]), which we accessed in January 2024. Samtools converted and sorted BAM files, with Seqkit creating alignment statistics. Chunk BAM was used to split aligned BAMs in, to chunks using the bundle_min_reads parameter (we used the default of 50,000). StringTie was then used to assemble the transcripts based on the aligned segments in the chunked BAM files. The resulting transcript GFF files were merged via Merge Chunks, and GFFCompare was used to compare query and reference annotations, merging and annotating records. The transcriptome FASTA files from the final GFFs were generated using Gffread. The reads from all samples were aligned with the final non-redundant transcriptome using Minimap2 in a splice-aware manner. Salmon (https://github.com/COMBINE-lab/salmon, accessed on 26 April 2024) was used for transcript quantification, giving gene and transcript counts as output. Because the Epi2me workflow did not have a time-course option, we then used this output to analyze differential expression as a time course experiment in DeSeq2, which we also used to create MA plots. We used the UniProt browser-based mapping (https://www.uniprot.org/id-mapping, accessed on 1 May 2024) to map UniProt gene names to Uniprot accession numbers, protein names, and GO terms. Raw and processed data were submitted to NCBI GEO (accession # GSE268152).

### 4.5. Bioinformatic Analysis

Heatmaps were generated based on log2FC (fold change) data using RStudio with the gplots package (https://cran.r-project.org/web/packages/gplots/index.html, accessed on 1 May 2024). To perform GO (gene ontology) enrichment and hierarchical clustering of selected genes at various stages of cluster root development, we employed the white lupin genome browser (https://www.whitelupin.fr) using the gene expression profile tools [35]. 

For analysis of the interactions among genes, we used sequences of our selected proteins as inputs for STRING (https://string-db.org) and selected the best hits based on E-values among *Arabidopsis thaliana* homologues. We then used the analysis tab, with a confidence setting of *p* > 0.1. 

To confirm cellular locations, we used DeepLoc 2.0 (https://services.healthtech.dtu.dk/services/DeepLoc-2.0, accessed on 1 May 2024) [49]. 

### 4.6. Validation by qRT-PCR 

A quantity of 1500 ng of total RNA for each sample (t0, t10, t15, t20) was treated with RNase-free DNase to eliminate genomic DNA contamination, then reverse-transcribed using iScript™ gDNA Clear cDNA Synthesis Kit (BioRad). 

Using the Reference Gene Selection Tool of the CFX Maestro software version 1.0 (BioRad), which utilizes the geNorm algorithm [37], we selected two reference genes (Histone H2A variant3 and proteasome endopeptidase complex) from a group of four candidates as the most stable in response to sucrose addition. 

Primers were designed using primer 3 [61] for the two reference genes and three target genes: codein 3-O demethylase, gibberellin-regulated protein, and bifunctional inhibitor/plant lipid transfer protein/seed storage helical-domain-containing protein, with amplicon sizes between 60–200 bp and no or low 3′ complementarity to avoid primer dimers. Primer sequences were as follows:

Histone H2A variant3 (Lalb_Chr11g0072021) forward: GAAGTTGCTATTGTTGATCTTGG, reverse: GCTGCATTGTTAATCACCTTTT; 

Proteasome endopeptidase complex (Lalb_Chr08g0243601) forward: TGCCTTTATGCCCTGCTGTA, reverse: CATCAAGCAACGCAAAACATG

Codeine 3-O-demethylase (Lalb_Chr20g0109071) forward: GGTGAGTTAGGTCCAGCATCT, reverse: ACTCCTGTTGTTTTGTACTGTGC; 

Bifunctional inhibitor (Lalb_Chr05g0225671) forward: TCAACTACTGTGGAAAGGGTGT, reverse: GCCAACGAGCTTCAGAAACC; 

Gibberellin regulated protein (Lalb_Chr02g0159351) forward: ACCTGGCAGTCTCAAAAGCT, reverse: TTTGTGGTACTGGGTCTGGC.

qPCR was performed using SYBR Green Supermix (BioRad) on a BioRad CFX96 instrument set to 30 s at 95 °C, followed by 40 cycles of 95 °C (15 s) and 60 °C (30 s). After amplification, a melt curve analysis was performed from 65 °C to 95 °C, with a 0.5 °C increment every 5 s. Cq values were called using the CFX Maestro Software (BioRad). Standard curves for all five genes were prepared, and amplification efficiency was determined. Relative gene expression was calculated using the ∆∆Cq method.

## Figures and Tables

**Table 1:** Sequence and mapping statistics.

cDNA Library– Biological Replicate	Number of Sequence Reads	Number of Mapped Sequences	Quality Mean	Length Range (after Trim)	Length Mean (after Trim)	Number of Mapped Genes
t0–rep 1	3,578,071	1,946,056	11	62–8182	700	22,960
t0–rep 2	3,820,082	1,903,893	11	61–7175	557	21,454
t0–rep 3	1,771,992	1,104,448	11	62–9278	748	21,427
t10–rep 1	3,118,626	2,063,375	11	61–8182	755	22,639
t10–rep 2	3,682,223	2,417,422	11	61–6392	774	23,322
t10–rep 3	2,230,808	1,296,886	11	61–11,327	699	22,243
t15–rep 1	2,177,882	1,043,045	11	61–6158	556	18,894
t15–rep 2	2,424,918	1,609,515	12	62–7296	765	22,278
t15–rep 3	4,093,433	2,059,972	11	62–11,327	620	24,655
t20–rep 1	3,996,371	2,656,449	11	62–8486	849	24,302
t20–rep 2	1,914,530	1,328,517	11	61–6542	843	21,933
t20–rep 3	2,736,983	1,450,390	11	62–12,545	578	22,544

**Table 2:** Seventeen genes up-regulated (log2FC ≥ 1.5, *p*-value ≤ 0.05) and two down-regulated (log2FC ≤ –1.5, *p*-value ≤ 0.05) at all three time points of sucrose exposure.

Gene ID, Description	Relevant GO Terms (Where Available)	10 min log2FC (*p*-Value)	15 min log2FC (*p*-Value)	20 min log2FC (*p*-Value)
Lalb_Chr03g0025221, Small auxin-up RNA (auxin-induced protein)	GO:0009733, response to auxin	5.1 (0.001)	4.0 (0.011)	4.3 (0.006)
Lalb_Chr16g0387181, Ovule protein	GO:0016020, membrane	4.6 (0.015)	5.3 (0.005)	4.2 (0.030)
Lalb_Chr25g0279531, Galactose-binding domain-containing protein		4.5 (0.003)	4.3 (0.005)	4.0 (0.009)
Lalb_Chr15g0076681, Uncharacterized protein		4.5 (0.006)	4.2 (0.012)	4.2 (0.011)
Lalb_Chr12g0198241, Bifunctional inhibitor/… domain-containing protein		4.4 (0.000)	4.4 (0.000)	3.7 (0.003)
Lalb_Chr22g0353301, WAT1-related (vacuolar auxin transport)	GO:0022857, transmembrane transporter	4.3 (0.004)	3.3 (0.029)	3.2 (0.034)
Lalb_Chr04g0254001, RNA recognition motif domain-containing	GO:0006397 (mRNA processing)	3.9 (0.004)	3.8 (0.007)	3.3 (0.017)
Lalb_Chr17g0342451, Glycosyltransferase	GO:0008194 (UDP-glycosyltransferase activity)	3.7 (0.014)	3.8 (0.012)	4.2 (0.005)
Lalb_Chr02g0158971, Transcription factor bHLH UPBEAT1	GO:0006355, regulation of DNA-templated transcription	3.4 (0.000)	3.0 (0.002)	3.3 (0.000)
Lalb_Chr20g0122831, 3,4-dihydroxy-2-butanone 4-phosphate synthase	GO:0009231 (riboflavin biosynthetic process)	3.2 (0.026)	3.0 (0.040)	2.9 (0.043)
Lalb_Chr17g0347051, Transcription factor bHLH family 61	GO:0006355, regulation of DNA-templated transcription	3.2 (0.037)	4.1 (0.007)	4.4 (0.003)
Lalb_Chr03g0033521, Expansin	GO:0009664 (plant-type cell wall organization)	3.0 (0.011)	2.6 (0.030)	2.5 (0.038)
Lalb_Chr16g0392421, Xyloglucan endotransglucosylase/hydrolase	GO:0071555 (cell wall organization)	3.0 (0.001)	2.6 (0.005)	2.5 (0.006)
Lalb_Chr20g0109071, Codeine 3-O-demethylase	GO:0008168, methyltransferase activity	2.9 (0.029)	2.9 (0.026)	4.0 (0.002)
Lalb_Chr08g0235731, Gibberellin-regulated protein	GO:0009744 (response to sucrose) GO:0009739 (response to gibberellin)	2.2 (0.010)	2.1 (0.014)	2.0 (0.020)
Lalb_Chr02g0159351, Gibberellin-regulated protein		2.1 (0.004)	2.0 (0.008)	1.6 (0.029)
Lalb_Chr08g0237751, Defensin	GO:0006952, defense response	1.9 (0.014)	1.7 (0.024)	1.6 (0.033)
Lalb_Chr19g0140251 Uncharacterized membrane protein		−6.6 (0.006)	−6.4 (0.007)	−6.6 (0.005)
Lalb_Chr05g0220091 Uncharacterized		−4.2 (0.015)	−3.8 (0.024)	−4.7 (0.008)

## Data Availability

Our data are freely accessible at NCBI GEO: GSE268152.
